# Surgical treatment for midportion Achilles tendinopathy: a systematic review

**DOI:** 10.1007/s00167-016-4062-9

**Published:** 2016-03-12

**Authors:** T. P. A. Baltes, R. Zwiers, J. I. Wiegerinck, C. N. van Dijk

**Affiliations:** Department of Orthopaedic Surgery, Academical Medical Center, University of Amsterdam, P.O. Box 22660, 1100 DD Amsterdam, The Netherlands

**Keywords:** Midportion Achilles tendinopathy, Endoscopic surgery, Minimally invasive surgery, Open surgery, Tenotomies

## Abstract

**Purpose:**

The aim of this study was to systematically evaluate the available literature on surgical treatment for midportion Achilles tendinopathy and to provide an overview of the different surgical techniques.

**Methods:**

A systematic review of the literature available in MEDLINE, EMBASE and the Cochrane database of controlled trials was performed. The primary outcome measure in terms of patient satisfaction and the secondary outcome measures that consisted of complication rate, pain score, functional outcome score and success rate were evaluated. The Downs & Black checklist and the Coleman methodology scale were used to assess the methodological quality of included articles.

**Results:**

Of 1090 reviewed articles, 23 met the inclusion criteria. The included studies reported on the results of 1285 procedures in 1177 patients. The surgical techniques were divided into five categories. Eleven studies evaluated open surgical debridement, seven studies described minimally invasive procedures, three studies evaluated endoscopic procedures, one study evaluated open gastrocnemius lengthening, and one study reported on open autologous tendon transfer. Results regarding patient satisfaction (69–100 %) and complication rate (0–85.7 %) varied widely.

**Conclusions:**

This study demonstrates the large variation in surgical techniques available for treatment of midportion Achilles tendinopathy. None of the included studies compared surgical intervention with nonsurgical or placebo intervention. Minimally invasive and endoscopic procedures yield lower complication rates with similar patient satisfaction in comparison with open procedures. Minimally invasive and endoscopic procedures might therefore prove to be the future of surgical treatment of Achilles midportion tendinopathy.

**Level of evidence:**

IV.

## Introduction

Midportion Achilles tendinopathy is a painful condition of the Achilles tendon located 2–7 cm proximal to the insertion on the calcaneus. Symptoms include pain, swelling and impaired performance. It is an over-use injury, often seen in athletes. The incidence varies from 0.2 % in the general population up to 9 % in recreational runners [12, 17].

Initially, treatment of midportion tendinopathy is nonsurgical. Various noninvasive treatment options have been described, including eccentric exercise, the use of orthoses and rest. Additionally, invasive nonsurgical techniques like extracorporeal shockwave therapy and platelet-rich plasma (PRP) injection are commonly used [39]. In approximately 25 % of the patients nonsurgical treatment fails to alleviate symptoms and surgical treatment is indicated [14].

Historically, surgical treatment was performed using an open procedure [13, 26, 32]. With new insights into the aetiology of tendinopathy, new surgical procedures were developed, resulting in a large variety of surgical techniques [19, 34]. Because of the high complication rates in open procedures, less invasive techniques gained popularity [16, 24, 29, 37].

Due to the variety of surgical techniques and the confusing terminology used to describe pathologies of the Achilles tendon, no study to date has provided a clear overview of the literature regarding the best surgical treatment for midportion Achilles tendinopathy. The aim of this study is to systematically evaluate the available literature on surgical treatment for midportion Achilles tendinopathy and to provide an overview of the different surgical techniques in terms of patient satisfaction and complication rate.

## Materials and methods

### Search strategy

**Fig. 1:**
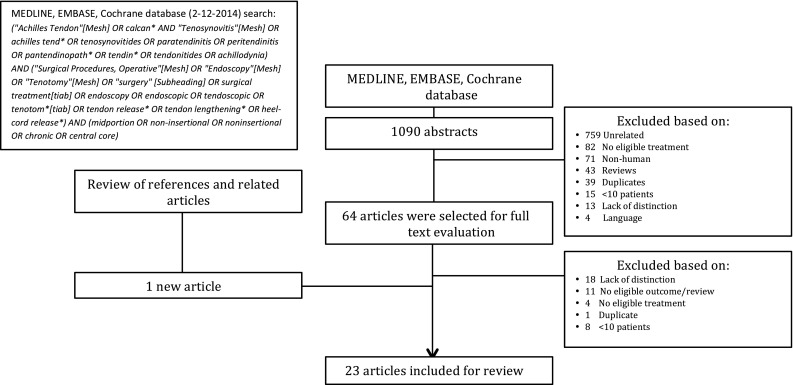
Search strategy

A systematic review of the literature was performed. In collaboration with a clinical librarian, the databases of MEDLINE, EMBASE and the Cochrane database for controlled trials were searched (Fig. 1).

### Inclusion criteria

Studies reporting on the results of surgical treatment for midportion Achilles tendinopathy in humans were included. In this study, the definition of midportion Achilles tendinopathy as described by van Dijk et al. [8] is used. Midportion Achilles tendinopathy is defined as symptoms of pain, swelling and impaired performance in combination with a histopathological diagnosis that includes, but is not limited to, the diagnosis of tendinosis 2–7 cm from the calcaneal insertion. Studies were eligible if they assessed the outcome measures of patient satisfaction or complication rate. Reviews and studies reporting on less than ten patients were excluded. Only studies written in English, French or German were considered eligible. Studies lacking clear distinction between midportion and insertional Achilles tendinopathy were excluded. Two authors (TB & RZ) performed title and abstract screening independently. Subsequently, these two authors individually performed full text selection. Disagreement during study selection was settled by discussion.

### Data extraction

Data on patient characteristics, surgical technique and outcome were extracted. Patient satisfaction was the primary outcome measure. Secondary outcome measures consisted of complication rate, functional outcome scores, pain scores and success rate. Complications were divided in minor and major (Table 1). Successful treatment was defined as an excellent or good outcome, or when patients were satisfied with the result.

**Table 1:** Classification of complications

Complications	
Major	Minor
AT rupture	Discomfort
Any reoperation	Superficial infections
Deep venous thrombosis	Minor wound problems
Reflex dystrophy	Scar tenderness/hypertrophy
Persisting neuralgia	Mild form of paraesthesia
Deep infections/major wound problems	Prolonged hospitalisation

### Methodological quality

Methodological quality of included studies was assessed using the Downs & Black checklist and the Coleman methodology scale [7, 9]. The Downs & Black checklist was designed to evaluate the methodological quality of randomised and nonrandomised studies. It consists of a 27-item checklist with a highest possible score of 33 points. The Coleman methodology scale was designed to evaluate the methodological quality of studies on Achilles tendinopathies. The checklist consists of two parts and has a maximum possible score of 100 points. Two authors (TB & JW) assessed the included studies independently on methodological quality. Correlations between success rate and both the Coleman scale and Downs & Black checklist were calculated.

### Data presentation and statistical analysis

Due to the heterogeneity of the data, results were not pooled. Outcome data were presented per study. Ranges of outcome were reported per type of surgical procedure. To calculate the correlations of nonparametric data, the Spearman’s rho correlation coefficient was used. Correlation with a *p* value <0.05 was considered significant.

## Results

After removal of duplicates, the search yielded 1090 articles. Based on title and abstract screening, 1026 articles were excluded (Fig. 1). The remaining 64 articles were eligible for full text evaluation. Forty-two articles were excluded after full text selection. Finally, after screening references of included articles and the related literature, one article was added [6]. A total of 23 studies were included in this study (Table 2; Appendix 1 in Table 5) [1–4, 6, 10, 11, 13, 15, 18, 20–24, 26–31, 33, 36].

**Table 2:** Overview of included studies

Study	*N* patients (*N* tendons)	Surgical technique	Coleman	D&B
Open debridement				
Benazzo et al. [4]	A. 20 (20)	A. Open surgery: multiple longitudinal tenotomies and excision degenerations	54	22
	B. 32 (32)	B. Open surgery: excision degeneration + soleus fibres transfer		
Johnston et al. [11]	A. 10 (10)	A. Open surgery: excision inflamed paratenon and decompression AT	29	7
	B. 7 (7)	B. Open surgery: excision inflamed paratenon, decompression AT + debridement degenerations		
Kvist and Kvist [13]	182 (201)	Open surgery: removal adhesions between paratenon, crural fascia and skin + excision thickened paratenon	27	14
Lohrer and Nauck [15]	A. 11 (15)	A. Open surgery: release AT + transachillear scarification	60	23
	B. 23 (24**)**	B. Open surgery: release AT + excision intratendinous lesions + transachillear scarification		
Maffulli et al. [18]	10 (10)	Open surgery: excision paratenon, multiple longitudinal tenotomies and excision of degenerations	39	10
Maffulli et al. [21]	86 (86)	Open surgery: excision paratenon, multiple longitudinal tenotomies + excision degenerations	69	14
Nelen et al. [26]	A. (93)	A. Open surgery: release AT (not ventrally) + excision inflamed paratenon	42	16
	B. (26)	B. Open surgery: debridement tendinosis		
	C. (24)	C. Open surgery: debridement tendinosis (reinforcement with turned-down tendon flap)		
Ohberg et al. [27]	24 (24)	Open surgery: excision hypertrophic paratenon and debridement of degenerations through a longitudinal incision	52	15
Paavola et al. [29]	A. 171 (171)	A. Open surgery: excision adhesions between crural fascia and paratenon	24	9
	B. 50 (50)	B. Open surgery: debridement intratendinous lesions		
Paavola et al. [28]	A. 16 (16)	A. Open surgery: excision adhesions between crural fascia and paratenon	56	14
	B. 26 (26)	B. Open surgery: excision adhesions between crural fascia and paratenon + debridement intratendinous lesions		
Sarimo and Orava [31]	24 (24)	Open surgery: fascial adhesiolysis and radiofrequency microtenotomy	36	20
Minimally invasive tendon stripping/tenotomies				
Alfredson et al. [2]	10 (10)	Minimally invasive: US–CD-guided release ventral AT	48	17
Alfredson [1]^a^	A. 15 (18)	A. Minimally invasive: US–CD-guided release ventral AT	41	17
	B. 16 (19)	B. Minimally invasive: US–CD-guided release ventral AT by use of a needle		
Alfredson [1]^a^	72 (88)	Minimally invasive: US–CD-guided release ventral AT	30	14
Alfredson et al. [3]	13 (13)	Minimally invasive: US–CD-guided release ventral AT	46	17
Calder et al. [6]	32 (34)	Minimally invasive: Release AT and excision plantaris tendon	59	21
Maffulli et al. [20]	39 (39)	Minimally invasive: multiple percutaneous longitudinal tenotomies	59	20
Naidu et al. [24]	26 (29)	Minimally invasive: circumferential AT release with a tracheal hook + peritendinous infusion of corticosteroids	40	17
Testa et al. [36]	63 (63)	Minimally invasive: multiple US-guided percutaneous longitudinal tenotomies	65	20
Endoscopic procedures				
Maquirriain [22]	24 (27)	Endoscopic: debridement paratenon + two longitudinal tenotomies	52	13
Pearce et al. [30]	11 (11)	Endoscopic: debridement paratenon + release of plantaris tendon	60	13
Steenstra and van Dijk [33]	16 (16)	Endoscopic: debridement paratenon + release of plantaris tendon	26	11
Open surgery: gastrocnemius lengthening				
Duthon et al. [10]	13 (15)	Open surgery: gastrocnemius lengthening	65	15
Open surgery: autologous tendon transfer				
Martin et al. [23]	44 (44)	Open surgery: excision AT + FHL transfer	54	21

Outlined are the number of included patients; reported surgical technique; and methodological quality

*AT* Achilles tendon, *US* ultrasound, *CD* colour doppler

^a^Same study comparing release of ventral AT with knife versus needle

### Population characteristics

The studies reported on a total of 1177 patients with 1285 affected tendons. 62.7 % of the patients was male. Seven studies did not report on gender distribution [11, 13, 26, 29, 30, 33, 36]. The weighted mean age was 40.2 years. The remaining seven studies did not adequately report on age [1, 10, 21, 26, 29, 33, 36].

### Surgical techniques

In this review, the techniques were roughly grouped into five types of procedures. Eleven studies reported open techniques as treatment of midportion Achilles tendinopathy [4, 11, 13, 15, 18, 21, 26–29, 31]. Seven studies evaluated minimally invasive procedures [1–3, 6, 20, 24, 36]. Three studies reported on endoscopic techniques [22, 30, 33], one study evaluated gastrocnemius lengthening by an open procedure [10], and one reported on a technique for open resection of the distal Achilles tendon, followed by interposition of FHL graft [23]. Techniques were classified as minimally invasive based on descriptions in the studies. In six of the included studies reporting minimally invasive techniques, incisions were smaller than 2 cm (Appendix 2 in Table 6).

### Open surgery

Eleven studies described the outcome of an open surgical approach [4, 11, 13, 15, 18, 21, 26–29, 31]. There is a large variation in techniques; however, each technique consisted of the release of the tendon (adhesiolysis, release or excision of the paratenon), debridement of degenerative tissue, longitudinal tenotomies or a combination of these (Table 2; Appendix 2 in Table 6). Four studies compared the results of release of the tendon with or without tenotomies, with a group of patients that additionally underwent debridement of degenerative tissue [11, 15, 28, 29]. One compared the results between a group of patients that underwent debridement without suturing or augmentation with a group in which the tendon was augmented using fibres of the soleus muscle [4]. In another study, three groups were distinguished: no debridement of the tendon was performed in group A. Group B and C consisted of patients with tendinosis and extensive tendinosis. Both groups were treated by excision of degenerations, followed by side-to-side suture for patients in group B or reconstruction with a turndown flap in group C [26].

None of the studies reported on patient satisfaction (Table 3). Complication rates were adequately reported in eight studies and ranged from 0 up to 85.7 % (Table 3) [4, 11, 13, 18, 21, 27, 29, 31]. Success rates varied from 73.1 up to 100 % (Table 4) [13, 15, 26–28, 31].

**Table 3:** Patient satisfaction and complication rate

Study	Postoperative satisfaction		Complications			
	Satisfied	Unsatisfied	Major	Minor	Total	Reoperations
Open debridement						
Benazzo et al. [4]			A. 1/20 (5 %)	A. 2/20 (10 %)	A. 3/20 (15 %)	A. 1/20 (5 %)
			B. 1/32 (3.1 %)	B. 3/32 (9.4 %)	B. 4/32 (12.5 %)	B. 1/32 (3.1 %)
Johnston et al. [11]			0/17 (0 %)	0/17 (0 %)	0/17 (0 %)	
Kvist and Kvist [13]			20/201 (10.0 %)	2/201 (1.0 %)	22/201 (10.9 %)	20/201 (10.0 %)
Lohrer and Nauck [15]						
Maffulli et al. [18]			6/14 (42.9 %)	6/14 (42.9 %)	12/14 (85.7 %)	6/14 (42.9 %)
Maffulli et al. [21]			8/86 (9.3 %)	23/86 (26.7 %)	31/86 (36.0 %)	8/86 (9.3 %)
Nelen et al. [26]			A+B+C. 12/143 (8.4 %)	A+B+C. 2/143 (1.4 %)	A+B+C. 14/143 (9.8 %)	A. 4/93 (4.3 %)
						B. 1/26 (3.8 %)
						C. 0/24 (0 %)
Ohberg et al. [27]			0/24 (0 %)	2/24 (8.3 %)	2/24 (8.3 %)	0/24 (0 %)
Paavola et al. [29]			A. 9/171 (5.3 %)	A.18/171 (10.5 %)	A. 27/171 (15.8 %)	A. 9/171 (5.3 %)
			B. 0/50 (0 %)	B. 1/50 (2 %)	B. 1/50 (2 %)	B. 0/50 (0 %)
Paavola et al. [28]			A+B. 2/42 (4.8 %)	A+B. 6/42 (14.3 %)	A+B. 8/42 (19.1 %)	A+B. 2/42 (4.8 %)
Sarimo and Orava [31]			1/24 (4.2 %)	1/24 (4.2 %)	2/24 (8.3 %)	
Totals			60/824 (7.3 %)	66/824 (8.0 %)	126/824 (15.3 %)	
Minimally invasive tendon stripping/tenotomies						
Alfredson et al. [2]	10/10 (100 %)		1/10 (10 %)	0/10 (0 %)	1/10 (10 %)	
Alfredson [1]^a^	A. 15/18 (83.3 %)^b^	A. 3/18 (16.7 %)^b^	A+B. 3/37 (8.1 %)	A+B. 0/37 (0 %)	A+B. 3/37 (8.1 %)	
	B. 15/19 (78.9 %)^b^	B. 4/19 (21.1 %)^b^				
Alfredson [1]^a^	81/88 (92.0 %)^b^	7/88 (8.0 %)^b^				
Alfredson et al. [3]	11/13 (84.6 %)	2/13 (15.4 %)				
Calder et al. [6]	22/32 (69 %)^c^	10/32 (31 %)^c^	0/34 (0 %)	1/34 (2.9 %)	1/34 (2.9 %)	
Maffulli et al. [20]						
Naidu et al. [24]	24/26 (92 %)^c^	2/26 (7.7 %)^c^	1/29 (3.4 %)	2/29 (6.9 %)	3/29 (10.3 %)	1/29 (3.4 %)
Testa et al. [36]			9/75 (12 %)	14/75 (18.6 %)	23/75 (30.6 %)	9/75 (12 %)
Totals			14/185 (7.6 %)	17/185(9.2 %)	31/185 (16.8 %)	
Endoscopic procedures						
Maquirriain [22]			2/27 (7.4 %)	0/27 (0 %)	2/27 (7.4 %)	
Pearce et al. [30]	8/11 (73 %)	3/11 (27 %)	0/11 (0 %)	0/11 (0 %)	0/11 (0 %)	0/11 (0 %)
Steenstra and van Dijk [33]			0/20 (0 %)	0/20 (0 %)	0/20 (0 %)	
Totals			2/58 (3.4 %)	0/58 (0 %)	2/58 (3.4 %)	
Open surgery: gastrocnemius lengthening						
Duthon et al. [10]	12/13 (92.3 %)^c^	1/13 (7.7 %)^c^	0/15 (0 %)	0/15 (0 %)	0/15 (0 %)	
Open surgery: autologous tendon transfer						
Martin et al. [23]	37/44 (86 %)	6/44 (13.8 %)	1/44 (2.3 %)	4/44 (9.1 %)	5/44 (11.4 %)	

^a^Same study comparing release of ventral AT with knife versus needle

^b^Study includes patients who received bilateral surgical treatment; outcome reported on individual tendons

^c^Study included patients who received bilateral surgical treatment; outcome reported on patient level

**Table 4:** Success rate

Study	Success rate	
	Successful	Unsuccessful
Open debridement		
Benazzo et al. [4]		
Johnston et al. [11]		
Kvist and Kvist [13]^b^	194/201 (96.5 %)	7/201 (3.5 %)
Lohrer and Nauck [15]^b^	15/15 (100 %)	
	23/24 (95.8 %)	1 (4.2 %)
Maffulli et al. [18]		
Maffulli et al. [21]		
Nelen et al. [26]^b^	A. 82/93 (88.2 %)	A. 11/93 (11.8 %)
	B. 19/26 (73.1 %)	B. 7/26 (26.9 %)
	C. 21/24 (87.5 %)	C. 3/24 (12.5 %)
Ohberg et al. [27]	22/24 (91.7 %)	2/24 (8.3 %)
Paavola et al. [29]		
Paavola et al. [28]	A. 16/16 (100 %)	
	B. 19/26 (73.1 %)	B. 7/26 (26.9 %)
Sarimo and Orava [31]	24/24 (100 %)	
Minimally invasive tendon stripping/tenotomies		
Alfredson et al. [2]	10/10 (100 %)	
Alfredson [1]^a,b^	A. 15/18 (83.3 %)	A. 3/18 (16.7 %)
	B. 15/19 (78.9 %)	B. 4/19 (21.1 %)
Alfredson [1]^a,b^	81/88 (92.0 %)	7/88 (8.0 %)
Alfredson et al. [3]	11/13 (84.6 %)	2/13 (15.4 %)
Calder et al. [6]^c^	22/32 (69 %)	10/32 (31 %)
Maffulli et al. [20]	30/39 (76.9 %)	9/39 (23.1 %)
Naidu et al. [24]^c^	24/26 (92 %)	2/26 (7.7 %)
Testa et al. [36]	47/63 (74.6 %)	16/63 (25.4 %)
Endoscopic procedures		
Maquirriain [22]^c^	24/24 (100 %)	
Pearce et al. [30]	8/11 (73 %)	3/11 (27 %)
Steenstra and van Dijk [33]		
Open surgery: gastrocnemius lengthening		
Duthon et al. [10]^c^	12/13 (92.3 %)	1/13 (7.7 %)
Open surgery: autologous tendon transfer		
Martin et al. [23]	37/44 (86 %)	6/44 (13.8 %)

^a^Same study comparing release of ventral AT with knife versus needle

^b^Study includes patients who received bilateral surgical treatment; outcome reported on individual tendons

^c^Study included patients who received bilateral surgical treatment; outcome reported on patient level

### Minimally invasive tendon stripping/tenotomies

Minimally invasive procedures were described in seven included studies [1–3, 6, 20, 24, 36]. Five articles described a technique to perform a release of the Achilles tendon [1–3, 6, 24] with additional excision of the plantaris tendon in one study [6]. The other two minimally invasive techniques described aimed to perform percutaneous longitudinal tenotomies with additional release of adhesions by infiltration performed in one study (Table 2, Appendix 2 in Table 6) [20, 36].

Patient satisfaction was reported in five studies and ranged from 69 up to 100 % (Table 3). [1–3, 6, 24]. Complications rates were adequately reported in four studies, ranging from 2.9 up to 30.6 % (Table 3) [2, 6, 24, 36]. Success rates varied from 69 up to 100 % (Table 4) [1–3, 6, 20, 24, 36].

### Endoscopic procedures

Three studies reported on the outcome of endoscopic procedures [22, 30, 33]. In all procedures, debridement of the paratenon was performed, with additional release of the plantaris tendon in two studies [30, 33] and additional longitudinal tenotomies in one study [22] (Table 2, Appendix 2 in Table 6).

Only one study reported on patient satisfaction; in this study 73 % of patients was satisfied (Table 3) [30]. Complications rates varied from 0 to 7.4 % (Table 3) [22, 30, 33]. Success rates varied from 73 up to 100 % (Table 4) [22, 30].

### Open surgery: gastrocnemius lengthening

One study evaluated gastrocnemius lengthening through an open procedure (Table 2, Appendix 2 in Table 6) [10]. Using this technique, a satisfaction rate of 92.3 % was achieved in combination with 0 % complications (Table 3). A success rate of 92.3 % was achieved (Table 4).

### Open surgery: autologous tendon transfer

One study reported on autologous tendon transfer [23]. The technique consisted of resection of the distal Achilles tendon, followed by interposition of the flexor hallucis longus (FHL) tendon (Table 2, Appendix 2 in Table 6).

The study reported that patient satisfaction was achieved in 86 % of the patients (Table 3). Complications occurred in 11.4 % of the patients (Table 3). A success rate of 86 % was achieved (Table 4).

### Methodological quality

The assessment of the methodological quality using the Downs & Black checklist and the Coleman methodological scale resulted in a median score of 12.0 (IQR 10.0–13.25) and 57.5 (IQR 40.0–64.25), respectively (Table 2).

There was no significant correlation between success rate and both Coleman score and Downs & Black score. However, a significant positive correlation was found between year of publication and Coleman score (rho: 0.53, *p* < 0.01), and year of publication and Downs & Black score (rho 0.66, *p* < 0.01).

## Discussion

The most important finding of the present study is the large variation in surgical techniques available for the treatment of midportion Achilles tendinopathy. Additionally, it demonstrates that minimally invasive and endoscopic procedures have lower complication rates with comparable patient satisfaction in comparison with open procedures. Due to low methodological quality of included studies in combination with large heterogeneity in population, used techniques and reported outcome measures, we refrained from data pooling.

Hitherto, one review evaluating surgical treatment of Achilles tendinopathy was performed [35]. In this study from 2001 a mean success rate of 77.4 % was reported [35]. Additionally, a negative correlation between success rate and methodological quality in studies on the topic of surgical treatment of tendinopathy was established [35]. In contrast to that review, we found no correlation between effectiveness and methodological quality. However, we found a positive correlation between year of publication and Coleman score, in line with a previous review on insertional tendinopathy [38]. This implicates that in current research on Achilles tendinopathy the methodological quality is improving.

Studies on open surgery reported inconsistent results regarding patient satisfaction and the complication rates varied widely. Particularly high complication rates were observed in studies evaluating the outcome of multiple tenotomies via an open procedure [4, 18, 21].

In general, good results regarding patient satisfaction and complication rates were reported in studies on minimally invasive surgery. Most of these studies reported on minimally invasive release of the Achilles tendon or minimally invasive longitudinal tenotomies of the Achilles tendon. One study evaluated the results of excision of the plantaris tendon [6]. In this study promising results were reported. However, more studies are needed to support these findings.

Minimally invasive procedures are assumed to have lower complication rates. Most studies evaluating minimally invasive techniques reported low complication rates (Table 3). However, a particularly high complication rate was observed in the study evaluating multiple percutaneous tenotomies [36].

Excellent results regarding functional outcome and postoperative pain were reported in three small series on endoscopic surgery. There were significant differences in the evaluated endoscopic procedures. Maquirriain et al. [22] reported the results of multiple endoscopic longitudinal tenotomies where in the other studies endoscopic release of the Achilles tendon in combination with release of the plantaris tendon was evaluated [30, 33]. Compared to the study by Pearce et al. a higher complication rate was observed in this study.

One study evaluating the results of gastrocnemius lengthening was included in this review [10]. Although promising results are reported, the study evaluated a mere 13 patients at final follow-up. A recent study reported less promising results regarding self-reported sport function in patients treated for Achilles tendinopathy with gastrocnemius lengthening [25].

One study on FHL transfer was included [23]. Good results were reported; however, a high complication rate was observed. The included retrospective case series reported on 44 patients with a mean age of 58.2 years. However, patients affected by midportion tendinopathy tend to be younger, and a wish for return to high-level sport is often present.

The most important limitation of this review is the limited level of evidence of included articles resulting in a high risk of bias. In addition, the lack of or incomplete reportage of our primary and secondary outcome measures made for an arduous comparison. In addition, the included studies used a large variety of heterogeneous outcome measures to measure functional outcome and pain. Therefore, caution should be taken when interpreting the results presented in this review. Another limitation is the confusing terminology used to describe the various pathological conditions of the Achilles tendon [8].

Despite the low level of evidence of the included study, this systematic review is of clinical significance [5]. This study is the first systematic review to compare surgical techniques for their effectiveness on patient satisfaction and complication rate. The study provides a comprehensive overview and clearly demonstrates the variation per surgical technique in outcome of patient satisfaction and complication rates.

We strongly encourage future studies to be conducted using validated instruments to assess pain and functional outcome. As often advocated, larger populations, prospective studies and long-term follow-up studies are needed to be conclusive on the best surgical treatment option of midportion Achilles tendinopathy.

In addition, no comparative study of surgical treatment and nonsurgical treatment or placebo intervention has been conducted. Therefore, no additional benefit of surgery over nonsurgical treatment, placebo intervention or wait-and-see policy has been established. Future research should therefore strive to compare surgical treatment with nonsurgical or placebo intervention.

Furthermore, as the exact pathophysiology of midportion tendinopathy is still poorly understood, current surgical techniques address a range of hypothetical abnormalities with varying results (Appendix 1 in Table 5). Future research should aim to determine whether the extent of the pathology influences the required surgical approach.

## Conclusion

The study provides a comprehensive overview of the current literature and clearly demonstrates the variation in outcome of patient satisfaction and complication rates. None of the included studies compared surgical treatment with nonsurgical treatment or placebo intervention. However, minimally invasive and endoscopic procedures result in lower complication rates compared to open surgical procedures. Minimally invasive and endoscopic procedures might therefore prove to be the future of surgical treatment of Achilles midportion tendinopathy.

## Appendix 1

See Table 5.

**Table 5:** Included studies

Study	Level of evidence	*N* patients (*N* tendons)	Gender (% F)	Mean age (years)	Duration of symptoms (months)	Mean F-U (months)	Diagnosis made by	Diagnosis	Surgical technique
Open debridement									
Benazzo et al. [4]	Level II	A. 20 (20)	A. 15 %	A. 41.6 ± 11.2	A. 9.14	A 6;.55.2 ± 14.4	A. History + Clinical + MRI	A. Midportion Achilles tendinopathy (= intratendinous lesions)	A. Open surgery: multiple longitudinal tenotomies and excision degenerations
		B. 32 (32)	B. 12.5 %	B. 43.2 ± 12.8	B. 8.91	B. 6; 47.2 ± 11.7	B. History + Clinical + MRI	B. Midportion Achilles tendinopathy (= intratendinous lesions)	B. Open surgery: excision degeneration + soleus fibres transfer
Johnston et al. [11]	Level IV	A. 10 (10)						A. Tenosynovitis	A. Open surgery: excision inflamed paratenon and decompression AT
		B. 7 (7)		B. 48 (33–58)				B. Chronic Achilles tendonitis + tenosynovitis^a^	B. Open surgery: excision inflamed paratenon, decompression AT + debridement degenerations
Kvist and Kvist [13]	Level IV	182 (201)		27.5 (12–53)	7.5 (2–120)			Calcaneal paratenonitis	Open surgery: removal adhesions between paratenon, crural fascia and skin + excision thickened paratenon
Lohrer and Nauck [15]	Level II	A. 11 (15)	A. 36.4 %	A. 48.4 (± 7.3; 39.0–58.0)		A. 3; 6; 12	A. Clinical + US-PD + MRI^a^	A. Isolated midportion Achilles tendinopathy	A. Open surgery: release AT + transachilllear scarification
		B. 23 (24)	B. 17.4 %	B. 50.5 (± 9.9; 23–66)		B. 3; 6; 12	B. Clinical + US-PD + MRI^a^	B. Midportion Achilles tendinopathy + central core lesions	B. Open surgery: release AT + excision intratendinous lesions + transachillear scarification
Maffulli et al. [18]	Level IV	10 (10)	20 %	34.8 (21–52)	87 (27–178)	35 (27–52)	Clinical + MRI^a^ + US^a^ + CT^a^	Achilles tendinopathy with central core degeneration, 2–6 cm area	Open surgery: excision paratenon, multiple longitudinal tenotomies and excision of degenerations
Maffulli et al. [21]	Level III	86 (86)	47.7 %		25.3 (12–52)	39.6 (30–60)	Clinical + US + Histological	Tendinopathy of the main body, 2–6 cm area	Open surgery: excision paratenon, multiple longitudinal tenotomies + excision degenerations
Nelen et al. [26]	Level IV	A. (93)						A. Peritendinitis	A. Open surgery: release AT (not ventrally) + excision inflamed paratenon
		B. (26)						B. Tendinosis	B. Open surgery: debridement tendinosis
		C. (24)						C. Extensive tendinosis	C. Open surgery: debridement tendinosis (reinforcement with turned-down tendon flap)
Ohberg et al. [27]	Level IV	24 (24)	29.2 %	43 (26–54)	>6	60 (31–82)	History + Clinical + US	Tendinosis, 2–6 cm level	Open surgery: excision hypertrophic paratenon and debridement of degenerations through a longitudinal incision.
Paavola et al. [29]	Level IV	A. 171 (171)			A. >3	A. 0.5; 1; 2.5; 12 (*N* = 28)	A. History + Clinical + US^a^	A. Peritendinitis	A. Open surgery: excision adhesions between crural fascia and paratenon
		B. 50 (50)			B. >3	B. 0.5; 1; 2.5; 12 (*N* = 28)	B. History + Clinical + US^a^	B. Tendinosis	B. Open surgery: debridement intratendinous lesions
Paavola et al. [28]	Level IV	A. 16 (16)	A. 25 %	A. 37 (±12)		A. 7	A. History + Clinical + MRI^a^ + US^a^	A. Tendinopathy: peritendinous adhesions	A. Open surgery: excision adhesions between crural fascia and paratenon
		B. 26 (26)	B. 34.6 %	B. 46 (±14)		B. 7	B. History + Clinical + MRI^a^ + US^a^	B. Tendinopathy: peritendinous adhesions + localised intratendinous lesion	B. Open surgery: excision adhesions between crural fascia and paratenon + debridement intratendinous lesions
Sarimo and Orava [31]	Level IV	24 (24)	50 %	54 (39–72)	14 (6–28)	30 (18–45)	History + Clinical + US	Chronic midportion Achilles tendinopathy	Open surgery: fascial adhesiolysis and radiofrequency microtenotomy
Minimally invasive tendon stripping/tenotomies									
Alfredson et al. [2]	Level I	10 (10)	40 %	45.2	23(6–60)	3; 6	Clinical + US–CD	Chronic painful midportion Achilles tendinosis	Minimally invasive: US–CD-guided release ventral AT
Alfredson [1]^b^	Level I	A. 15 (18)	A. 33.3 %	A. 46 (31–67)	A. 74 (7–240)		A. Clinical + US–CD	A. Midportion Achilles tendinosis	A. Minimally invasive: US–CD-guided release ventral AT.
		B. 16 (19)	B. 37.5 %	B. 47 (31–76)	B. 82 (5–240)		B. Clinical + US–CD	B. Midportion Achilles tendinosis	B. Minimally invasive: US–CD-guided release ventral AT by use of a needle.
Alfredson [1]^b^	Level IV	72 (88)	36.1 %		>3		Clinical + US–CD	Midportion Achilles tendinosis	Minimally invasive: US–CD-guided release ventral AT.
Alfredson et al. [3]	Level IV	13 (13)	46.2 %	53 (34–70)	>6	6	Clinical + US–CD	Midportion Achilles tendinopathy	Minimally invasive: US–CD-guided release ventral AT.
Calder et al. [6]	Level IV	32 (34)	31.3 %	27.2 (19–42)		22.1 (12–48)	Clinical + MRI	Noninsertional Achilles tendinopathy (= paratendinitis + tendinosis^a^)	Minimally invasive: release AT and excision plantaris tendon
Maffulli et al. [20]	Level IV	39 (39)	23.1 %	45 (34–63)	13 (6–55)	204 (180–264)	Clinical + US	Midportion Achilles tendinopathy + paratendinopathy^a^	Minimally invasive: multiple percutaneous longitudinal tenotomies
Naidu et al. [24]	Level IV	26 (29)	68.8 %	53 (38–66)		13 (6–31)	Clinical + radiograph + MRI^a^ + US^a^	Noninsertional Achilles tendinopathy	Minimally invasive: circumferential AT release with a tracheal hook + peritendinous infusion of corticosteroids
Testa et al. [36]	Level IV	63 (63)				51 (±8.2)	History + Clinical + MRI^a^ + US	Chronic Achilles tendinopathy + paratendinopathy^a^	Minimally invasive: multiple US-guided percutaneous longitudinal tenotomies
Endoscopic procedures									
Maquirriain [22]	Level IV	24 (27)	50 %	45.5 (±8.9)	>6	92.4 (60–168)	History + Clinical + MRI	Chronic midportion Achilles tendinopathy	Endoscopic debridement paratenon + two longitudinal tenotomies
Pearce et al. [30]	Level IV	11 (11)		36.5 (24–55)	13 (3–36)	30 (24–39)	Clinical + MRI^a^ + US^a^	Noninsertional tendinopathy (= paratendinopathy + intratendinous disease)	Endoscopic debridement paratenon + release of plantaris tendon
Steenstra and van Dijk [33]	Level IV	16 (16)			>24	72 (24–84)		Noninsertional tendinopathy + paratendinopathy.	Endoscopic debridement paratenon + release of plantaris tendon
Open surgery: gastrocnemius recession									
Duthon et al. [10]	Level IV	13 (15)	23.1 %			12; 24	History + Clinical + MRI^a^	Noninsertional Achilles tendinopathy (= peritendonitis + tendinosis)	Open surgery: gastrocnemius recession
Open surgery: autologous tendon transfer									
Martin et al. [23]	Level IV	44 (44)	59 %	58.2 (±10.1)		40.8 (±22.8)	Clinical + Radiograph + MRI	Chronic degenerative Achilles tendinosis, 2–6 cm area	Open surgery: excision AT + FHL transfer

Outlined are level of evidence; patient demographics; duration of follow-up (F-U); reported surgical technique and methodological quality

*AT* Achilles tendon, *US* ultrasound, *CD* color doppler

^a^Only in percentage of patients

^b^Same study comparing release of ventral AT with knife versus needle

## Appendix 2

See Table 6.

**Table 6:** Surgical techniques

	*N* of procedures	*N* of surgeons	Position	Approach	Description
Open debridement					
Benazzo et al. [4]	A. 20	A. 1	A. Prone	A. Lateral longitudinal incision	A. Two–three longitudinal tenotomies and excision of degenerated areas (not sutured)
	B. 32	B. 2	B. Prone	B. Lateral longitudinal incision	B. Excision of the degenerations through a longitudinal tenotomy after which a muscle bundle of the soleus muscle was bluntly dissected and distally left attached. The proximal end of the muscle bundle was anchored into the longitudinal incision with absorbable sutures
Johnston et al. [11]	A. 10			A. Medial longitudinal incision	A. Resection of thickened peritenon after which a one cm portion was resected and the AT was decompressed
	B. 7			B. Medial longitudinal incision	B. Resection of thickened peritenon after which a one cm portion was resected. If the AT was thickened or swollen, a longitudinal incision was made and degenerations were debrided
Kvist and Kvist [13]	201		Prone	Lateral incision (7 cm)	Fascial incision, after which adhesions between the paratenon and crural fascia and the crural fascia and the skin were removed. Thickened paratenon was excised (not sutured)
Lohrer and Nauck [15]	A. 15	A. 1		A. Transverse incision (4 cm) + s.o.s. expanded longitudinally medial or lateral, creating a Z- or L-shape	A. The AT was released, after which transachillear scarification, parallel to the fibres, was performed with a surgical scalpel
	B. 24	B. 1		B. Transverse incision (4 cm) + s.o.s. expanded longitudinally medial or lateral, creating a Z- or L-shape	B. The AT was released, after which a longitudinal incision was made to excise degenerated lesions. The AT was reconstructed with sutures. With a surgical scalpel transachillear scarification was performed, parallel to the fibres
Maffulli et al. [18]	10	1	Prone	Medial longitudinal incision	The paratenon was excised and suspicious areas were explored by three–five longitudinal tenotomies and degenerations were excised (not sutured)
Maffulli et al. [21]	86		Prone	Medial or lateral curvilinear longitudinal incision (10-12 cm)	The paratenon was excised and suspicious areas were explored by three–five longitudinal tenotomies and degenerations were excised (not sutured)
Nelen et al. [26]	A. 93			A. Medial longitudinal incision	A. Incision of crural fascia and paratenon, after which the medial, lateral and dorsal aspect of the AT were released (no circular dissection, ventral side AT left untouched). Hypertrophic paratenon was excised
	B. 26			B. Medial longitudinal incision	B. Debridement tendinosis (sutured side to side)
	C. 24			C. Medial longitudinal incision	C. Extensive debridement tendinosis, after which the AT was reinforced with a rectangular flap of lateral or medial tendon aponeurosis, turned down on itself and sutured in the defect with resorbable sutures
Ohberg et al. [27]	24			Lateral longitudinal incision	Hypertrophic paratenon was excised, and a longitudinal incision was made to debride degenerations (sutured side to side)
Paavola et al. [29]	A. 171	A. 1	A. Prone	A. Lateral longitudinal incision	A. Fascial incision, after which adhesions between the paratenon and the crural fascia were excised
	B. 50	B. 1	B. Prone	B. Lateral longitudinal incision	B. Fascial incision, after which adhesions between paratenon and crural fascia were removed and a longitudinal incision was made to excise intratendinous lesions (sutured side to side)
Paavola et al. [28]	A. 16	A. 2	A. Prone	A. Lateral longitudinal incision	A. Fascial incision, after which adhesions between paratenon and crural fascia were removed
	B. 26	B. 2	B. Prone	B. Lateral longitudinal incision	B. Fascial incision, after which adhesions between paratenon and crural fascia were removed and a longitudinal incision was made to excise intratendinous lesions (sutured side to side)
Sarimo and Orava [31]	24			Medial or Lateral longitudinal incision (3–5 cm)	Fascial incision, after which adhesions between paratenon and crural fascia were removed and multiple radiofrequency microtenotomies were performed
Minimally invasive tendon stripping/tenotomies					
Alfredson et al. [2]	10			Lateral longitudinal incision	US–CD-guided dissection of the AT from the ventral soft tissue by use of a knife followed by haemostasis with diathermia
Alfredson [1]^a^	A. 18		A. Prone	A. Lateral longitudinal incision (1–2 cm)	A. US–CD-guided dissection of the AT from the ventral soft tissue by use of a knife followed by haemostasis with diathermia
	B. 19		B. Prone	B. Medial or lateral needle insertion	B. US–CD-guided release of the AT from the ventral soft tissue by use of a needle
Alfredson [1]^a^	88		Prone	Lateral longitudinal incision (1–2 cm)	US–CD-guided dissection of the AT from the ventral soft tissue by use of a knife, followed by haemostasis with diathermia
Alfredson et al. [3]	13			Medial longitudinal incision (1–2 cm)	US–CD-guided dissection of the AT from the ventral soft tissue by use of a knife followed by haemostasis with diathermia
Calder et al. [6]	34			Medial incision (2–3 cm)	Release of the AT, after which the plantaris tendon was released from the AT and transected distally. The proximal end of the plantaris tendon is sectioned at the musculo-tendinous junction and delivered through a stab incision
Maffulli et al. [20]	39	1	Prone	Five stab incisions: 2 medial; 1 central; 2 lateral	Multiple US-guided percutaneous longitudinal tenotomies were created through five stab incisions
Naidu et al. [24]	29		Prone	Midline longitudinal incision (1–2 cm)	A blunt tracheal hook was passed up and down the AT to perform adhesiolysis. After closure of the paratenon, corticosteroids were infused peritendinously
Testa et al. [36]	63		Prone	Stab incision central over degeneration	Adhesiolysis by 0.5 % carbocaine infiltration. Next, six US-guided percutaneous longitudinal tenotomies through one incision, three up- and three downwards, varying 45° were performed
Endoscopic procedures					
Maquirriain [22]	27	1	Prone	Two midline portals	Endoscopic debridement of paratenon and release of the crural fascia were performed. Thereafter, two longitudinal tenotomies were performed
Pearce et al. [30]	11		Prone	Proximal portal medial + distal portal lateral	Endoscopic debridement of paratenon and release of the plantaris tendon were performed
Steenstra and van Dijk [33]	16		Prone	Proximal portal medial + distal portal lateral	Endoscopic debridement of paratenon and release of the plantaris tendon were performed
Open surgery: gastrocnemius lengthening					
Duthon et al. [10]	15	1	Supine	Medial incision (5 cm)	The gastrocnemius muscle was separated from the soleus muscle by blunt dissection after which the gastrocnemius muscle was cut transversally (not sutured)
Open surgery: autologous tendon transfer					
Martin et al. [23]	44		Supine	Medial longitudinal incision (10 cm)	The distal four–six cm of the AT was excised after which the FHL was harvested and interpositioned (secured proximally with a Pulvertaft weave, distally a tunnel is drilled in the calcaneus and the FHL is secured with an interference screw or reflected onto self and sutured)

Outlined are number of procedures; number of involved surgeons; positioning; approach and used surgical technique

*AT* Achilles tendon, *US* ultrasound, *CD* color doppler

^a^Same study comparing release of ventral AT with knife versus needle

## Appendix 3

See Table 7.

**Table 7:** Functional outcome and pain score

	Functional outcome measures		Pain scores		Result at follow-up	
Study	Preoperative	At follow-up	Preoperative	At follow-up	Successful	Unsuccessful
Open debridement						
Benazzo et al. [4]	A. AOFAS: 72.0 ± 11.6	A. AOFAS (6 months): 93.7 ± 5.0^a^ (55.2 months): 89.8 ± 3.8				
	A. VISA-A: 53.4 ± 14.7	A. VISA-A (6 months): 93.3 ± 5.3^a^ (55.2 months): 88.7 ± 5.4				
	A. TEGNER: 6 ± 1.2	A. TEGNER (55.2 months): 6 ± 0.9				
	B. AOFAS: 69.0 ± 10.2	B. AOFAS (6 months): 98.5 ± 3.4^a^ (47.2 months): 95.1 ± 5.1				
	B. VISA-A: 51.9 ± 15.4	B. VISA-A (6 months): 96.5 ± 5.4^a^ (47.2 months): 94.4 ± 5.8				
	B. TEGNER: 6 ± 1.4	B. TEGNER (47.2 months): 6 ± 1.1				
Johnston et al. [11]						
Kvist and Kvist [13]^d^					194/201 (96.5 %)	7/201 (3.5 %)
Lohrer and Nauck [15]^d^	A. VISA-A: 44.2 ± 5.5 (16–66)	A. VISA-A (3 months): 51.4 ± 20.4 (12–86) (6 months): 76.2 ± 18.5 (48–99)^a^ (12 months): 86.3 ± 8.8 (73–97)^a^			A. 15/15 (100 %)	
	B. VISA-A: 37.0 ± 17.6 (10–79)	B. VISA-A (3 months): 57.0 ± 23.2 (12–97) (6 months): 81.0 ± 15.8 (50–99)^a^ (12 months) 90.3 ± 10.6 (62–100)^a^			B. 23/24 (95.8 %)	B. 1/24 (4.2 %)
Maffulli et al. [18]						
Maffulli et al. [21]						
Nelen et al. [26]^d^					A. 82/93 (88.2 %)	A. 11/93 (11.8 %)
					B. 19/26 (73.1 %)	B. 7/26 (26.9 %)
					C. 21/24 (87.5 %)	C. 3/24 (12.5 %)
Ohberg et al. [27]					22/24 (91.7 %)	2/24 (8.3 %)
Paavola et al. [29]						
Paavola et al. [28]					A. 16/16 (100 %)	
					B. 19/26 (73.1 %)	B. 7/26 (26.9 %)
Sarimo and Orava [31]			NPSAO 5.0 (3–7)	NPSAO: 0.7 (0–4)^a^	24/24 (100 %)	
				VAS rest: 0 mm		
				VAS daily living: 1.4 mm (0–20 mm)		
				VAS athletic activity: 6.0 mm (0–40 mm)		
Minimally invasive tendon stripping/tenotomies						
Alfredson et al. [2]			VAS (3 months): 75 mm	VAS (3 months): 21 mm^a^	10/10 (100 %)	
Alfredson [1]^b,d^			A. VAS AT-loading: 69 mm (40–92 mm)	A. VAS AT-loading: 6 mm (0–38 mm)^a^	A. 15/18 (83.3 %)	A. 3/18 (16.7 %)
			B. VAS AT-loading: 75 mm (40–99 mm)	B. VAS AT-loading: 2 mm (0–15 mm)^a^	B. 15/19 (78.9 %)	B. 4/19 (21.1 %)
Alfredson [1]^b,d^					81/88 (92.0 %)	7/88 (8.0 %)
Alfredson et al. [3]			VAS AT-loading: 76 mm (55–98 mm)	VAS AT-loading: 7 mm (0–23 mm)	11/13 (84.6 %)	2/13 (15.4 %)
Calder et al. [6]^e^	FAOS: 333 (321–345)	FAOS: 449 (431–468)	VAS: 58 mm (95 % CI 5.4–6.3)	VAS: 8 mm (95 % CI 0.3–1.3)^a^	22/32 (69 %)	10/32 (31 %)
Maffulli et al. [20]		VISA-A: 78.5 (51–94)			30/39 (76.9 %)	9/39 (23.1 %)
Naidu et al. [24]^e^	Puddu scale: 4.9/6	Puddu scale: 1.7/6	VAS: 87 mm	VAS: 24 mm^a^	24/26 (92 %)	2/26 (7.7 %)
Testa et al. [36]					47/63 (74.6 %)	16/63 (25.4 %)
Endoscopic procedures						
Maquirriain [22]^e^	VISA-A (*N* = 15): 37.06 ± 4.99	VISA-A (*N* = 15): 97.55 ± 12.11^a^		VAS 2.2 mm ± 11.5 mm	24/24 (100 %)	
	ATSS-score: 32.66 ± 13.15	ATSS-score: 97.25 ± 12.31^a^				
		PGART: 0.25 ± 0.71				
Pearce et al. [30]	AOFAS: 68 (51–82)	AOFAS: 92 (74–100)^a^	AOS-pain: 28 %	AOS-pain: 8 %^a^	8/11 (73 %)	3/11 (27 %)
	AOS-disability: 38 %	AOS-disability: 10 %^a^				
	SF-36: 76	SF-36: 87				
Steenstra and van Dijk [33]						
Open surgery: gastrocnemius lengthening						
Duthon et al. [10]^e^	AOFAS: 71 (67–73)^c^	AOFAS (12 months *N* = 17^d^): 100 (90–100)^a,c^			12/13 (92.3 %)	1/13 (7.7 %)
	FFI: 39 (25–45)^c^	FFI (12 months *N* = 17^d^): 12 (10–18)^a,c^; (24 months *N* = 17^d^): 12 (10–19)^a,c^				
	SF-12 physical: 36 (33–44)^c^	SF-12 physical (12 months *N* = 17^b^): 51 (42–56)^a,c^; (24 months *N* = 17^d^): 51 (46–56)^a,c^				
Open surgery: autologous tendon transfer						
Martin et al. [23]		AOFAS (*N* = 19): 91.6 ± 7.7		VAS: 15 mm (±20 mm)	37/44 (86 %)	6/44 (13.8 %)

^a^Statistically significant (*p* < 0.05)

^b^Same study divided into two separate studies

^c^Median instead of mean

^d^Study includes patients who received bilateral surgical treatment; outcome reported on individual tendons

^e^Study included patients who received bilateral surgical treatment; outcome reported on patient level

## Appendix 4

See Table 8.

**Table 8:** Success rate

Study	Result at F-U						Success rate	
	Satisfied	Unsatisfied	Excellent	Good	Fair	Poor	Successful	Unsuccessful
Open debridement								
Benazzo et al. [4]								
Johnston et al. [11]								
Kvist and Kvist [13]^b^			169/201 (84.1 %)	25/201 (12.4 %)		7/201 (3.5 %)	194/201 (96.5 %)	7/201 (3.5 %)
Lohrer and Nauck [15]^b^			A. 15/15 (100 %)				A. 15/15 (100 %)	
			B. 23/24 (95.8 %)		B. 1/24 (4.2 %)		B. 23/24 (95.8 %)	B. 1/24 (4.2 %)
Maffulli et al. [18]								
Maffulli et al. [21]								
Nelen et al. [26]^b^			A. 54/93 (58.1 %)	A. 28/93 (30.1 %)	A. 8/93 (8.6 %)	A. 3/93 (3.2 %)	A. 82/93 (88.2 %)	A. 11/93 (11.8 %)
			B. 15/26 (57 %)	B. 4/26 (15.4 %)	B. 4/26 (15.4 %)	B. 3/26 (11.5 %)	B. 19/26 (73.1 %)	B. 7/26 (26.9 %)
			C. 12/24 (50 %)	C. 9/24 (37.5 %)	C. 2/24 (8.3 %)	C. 1/24 (4.2 %)	C. 21/24 (87.5 %)	C. 3/24 (12.5 %)
Ohberg et al. [27]			12/24 (50 %)	10/24 (41.7 %)		2/24 (8.3 %)	22/24 (91.7 %)	2/24 (8.3 %)
Paavola et al. [29]								
Paavola et al. [28]			A. 5/16 (31.3 %)	A. 11/16 (68.8 %)			A. 16/16 (100 %)	
			B.7/26 (26.9 %)	B.12/26 (46.2 %)	B. 1/26 (3.8 %)	B. 6/26 (23.1 %)	B. 19/26 (73.1 %)	B. 7/26 (26.9 %)
Sarimo and Orava [31]			14/24 (58.3 %)	10/24 (41.7 %)			24/24 (100 %)	
Minimally invasive tendon stripping/tenotomies								
Alfredson et al. [2]	10/10 (100 %)						10/10 (100 %)	
Alfredson [1]^a,b^	A. 15/18 (83.3 %)	A. 3/18 (16.7 %)					A. 15/18 (83.3 %)	A. 3/18 (16.7 %)
	B. 15/19 (78.9 %)	B. 4/19 (21.1 %)					B. 15/19 (78.9 %)	B. 4/19 (21.1 %)
Alfredson [1]^a,b^	81/88 (92.0 %)	7/88 (8.0 %)					81/88 (92.0 %)	7/88 (8.0 %)
Alfredson et al. [3]	11/13 (84.6 %)	2/13 (15.4 %)					11/13 (84.6 %)	2/13 (15.4 %)
Calder et al. [6]^c^	22/32 (69 %)	10/32 (31 %)					22/32 (69 %)	10/32 (31 %)
Maffulli et al. [20]			30/39 (76.9 %)		9/39 (23.1 %)		30/39 (76.9 %)	9/39 (23.1 %)
Naidu et al. [24]^c^	24/26 (92 %)	2/26 (7.7 %)					24/26 (92 %)	2/26 (7.7 %)
Testa et al. [36]			35/63 (55.6 %)	12/63 (19.0 %)	8/63 (12.7 %)	8/63 (12.7 %)	47/63 (74.6 %)	16/63 (25.4 %)
Endoscopic procedures								
Maquirriain [22]^c^			20/24 (85.1 %)	4/24 (14.9 %)			24/24 (100 %)	
Pearce et al. [30]	8/11 (73 %)	3/11 (27 %)					8/11 (73 %)	3/11 (27 %)
Steenstra and van Dijk [33]								
Open surgery: gastrocnemius lengthening								
Duthon et al. [10]^c^	12/13 (92.3 %)	1/13 (7.7 %)					12/13 (92.3 %)	1/13 (7.7 %)
Open surgery: autologous tendon transfer								
Martin et al. [23]	37/44 (86 %)	6/44 (13.8 %)					37/44 (86 %)	6/44 (13.8 %)

Outlined are patient satisfaction; postoperative result; and success rate

^a^Same study comparing release of ventral AT with knife versus needle

^b^Study includes patients who received bilateral surgical treatment; outcome reported on individual tendons

^c^Study included patients who received bilateral surgical treatment; outcome reported on patient level
